# Recurrent acinic cell carcinoma of the parotid gland with lateral skull base invasion: Case report and discussion of the literature

**DOI:** 10.1002/ccr3.7512

**Published:** 2023-07-17

**Authors:** Pietro De Luca, Matteo Calvanese, Angelo Camaioni, Giorgio Iaconetta, Maurizio Iemma

**Affiliations:** ^1^ Otolaryngology Department San Giovanni‐Addolorata Hospital Rome Italy; ^2^ Department of Otorhinolaryngology University Hospital “San Giovanni di Dio e Ruggi d'Aragona” Salerno Italy; ^3^ Neurosurgery Unit, Department of Medicine, Surgery and Dentistry University Hospital “San Giovanni di Dio e Ruggi d'Aragona” University of Salerno Salerno Italy

**Keywords:** acinic cell carcinoma, lateral skull base, parotid gland, salivary glands

## Abstract

Medical records of a 76‐year‐old woman with a recurrent acinic cell carcinoma of the left parotid gland with lateral skull base invasion were reviewed. She underwent subtotal petrosectomy followed by radiation therapy. After surgery, she remained disease‐free for more than 16 months.

## 1 INTRODUCTION

Acinic cell carcinoma (AciCC) is an uncommon malignant epithelial neoplasm of the salivary glands typically arising in the parotid gland (85% of cases)^1^, ^2^; it comprises 7%–15% of all malignant tumors of the major salivary glands, and 5% of those of parotid gland.^3^, ^4^

At first classified as a “benign adenoma” (or “acinic cell tumor”) by Goodwin et al.^5^ in 1890, Buxton et al.^6^ recognized it as a malignant tumor in 1953, and recently WHO re‐classified AciCC as malignant carcinoma.^7^

Women are affected only slightly more frequently than men, with a slight peak in the fifth and sixth decades; patients ranging from young children to centenarians.^8^ Possible causes of AciCC include previous radiation exposure and familiar predisposition. There are no distinctive radiological patterns (MRI, CT, ultrasound imaging), and it commonly occurs as an indolent, well‐defined solid cystic mass.

Complete surgical excision of the primary tumor offers the best opportunity for cure.^9^ Radiation therapy should be considered in some selected cases (positive surgical margins, multiple positive lymph nodal involvement, perineural invasion, high histologic grade, stage T3 or T4). However, the role of radiotherapy and chemotherapy remains unclear.^10^ To the best of our knowledge, descriptions of parotid AciCC with skull base invasion have been limited only to 12 case reports.^11^, ^12^, ^13^

The aim of this paper is to report a case of recurrent AciCC of the parotid gland with lateral skull base invasion and to review the existing literature about this rare neoplasm.

## 2 CASE REPORT

A 74‐year‐old woman was admitted in June 2018 to our Otolaryngology Department with a hard, indolent and fixed left parotid mass. The patient reported onset of symptoms 3 months earlier. Physical examination showed no facial paralysis. Ten years before she had a left breast cancer and actually she took no medications. She was not an active smoker with no alcohol or drug use.

A CT of the neck after contrast showed a 2 × 3 × 3 cm heterogeneously enhanced mass in the deep lobe of the left parotid gland, without extraparotid extension. No lymph node abnormalities were observed.

An ultrasound guided fine needle aspiration biopsy (FNAB) was performed and the citology suggested a poorly differentiated basal cells neoplasm (cT2N0M0). Consequently, the Head and Neck Tumor Board recommended a total surgical excision.

The patient underwent a total left parotidectomy with facial nerve monitoring and facial nerve preservation. She was discharged 5 days after surgery and recovered without incident. A final pathology showed an AciCC of the left parotid gland with low degree of malignancy, without perineural invasion and with free resection margins (pT3N0M0).

As a result, the Head and Neck Tumor Board, supported by the last medical literature, did not recommend complementary radiation therapy.

She reported at the Head and Neck Department in February 2019, due to otalgia and otorrhea from the left ear and complete facial paralysis; the physical examination showed a swelling at the site of the previous surgery.

**FIGURE 1:**
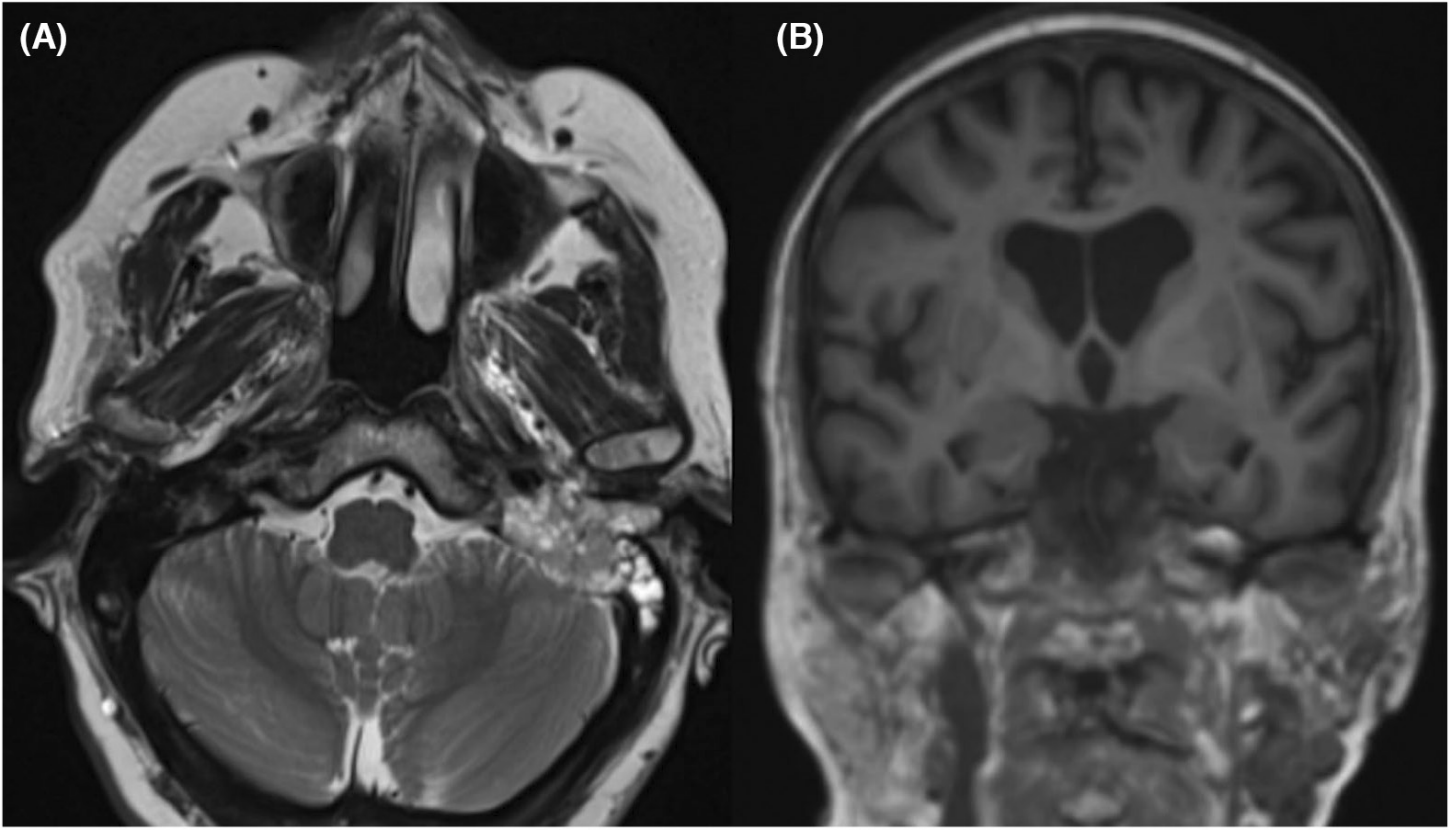
Pre‐operative CT Scans; (A) Axial CT Section; the tumor involved the left pontocerebellar angle; (B) Coronal CT Section; the neoplasm involved the left petrous bone, the left pontocerebellar angle and the fibers of VII, VIII, IX, X, XI, XII cranial nerves.

A CT of the neck with intravenous contrast, showed many colliquative areas in the parotid area, with lytic tissue extended to the external auditory canal, the tympanic cavity, and to the mastoid, with infiltration of the sigmoid sinus and erosion of the labyrinthine block up to the internal auditory canal and to the pontocerebellar angle (Figure 1).

A FNAB was performed and citology revealed a recurrence of AciCC of the parotid gland.

The patient underwent a lateral skull base resection. A lateral approach to the skull base by pre‐sigmoid‐retrolabirinthine has been performed. The posterior and lateral semicircular canals have been skeletonized. The facial nerve was exposed in its second genu and the rest of the nerve was encased by the tumor; therefore, it was not possible to preserve it. The jugular bulb, very thin, started bleeding during the delicate dissection of the tumor, and bleeding was stopped by using Gelfoam. By introducing the endoscope, the clivus and the abducens nerve were on the view. A total excision of the tumor up to the left pontocerebellar angle^14^, ^15^ and a left modified neck radical dissection (levels I–V) with external carotid artery ligation were performed. Surgery was performed by using microscope and endoscope, extremely useful in order to check all the corners of the tumor bed, to be sure that a total removal had been performed. The defect of the dura mater was repaired by a dura patch, and the petromastoid cavity was filled by using abdominal fat and fibrin glue, in order to avoid cerebrospinal fluid distulas. Spinal drainage was not inserted.

**FIGURE 2:**
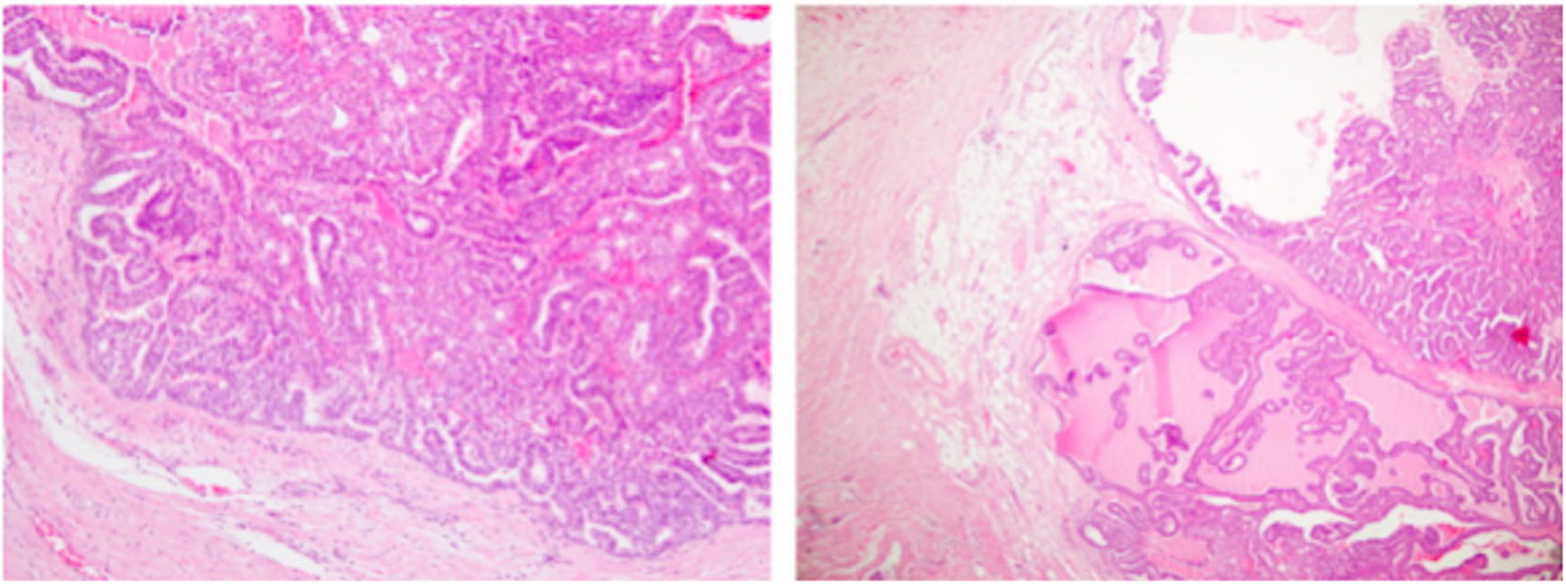
Post‐operative hystologic examination showed an acinic cell carcinoma.

The histologic examination was in favor of a recurrence of AciCC (Figure 2) (pT4N1M0).

Postoperative outcome was good and she was discharged from hospital 2 weeks after surgery, with recommendation to start treatment with neutron radiotherapy at a total dose of 66 Gy, without chemotherapy. A CT scan, performed 3 months and 6 months after radiation therapy, showed stable disease.

**FIGURE 3:**
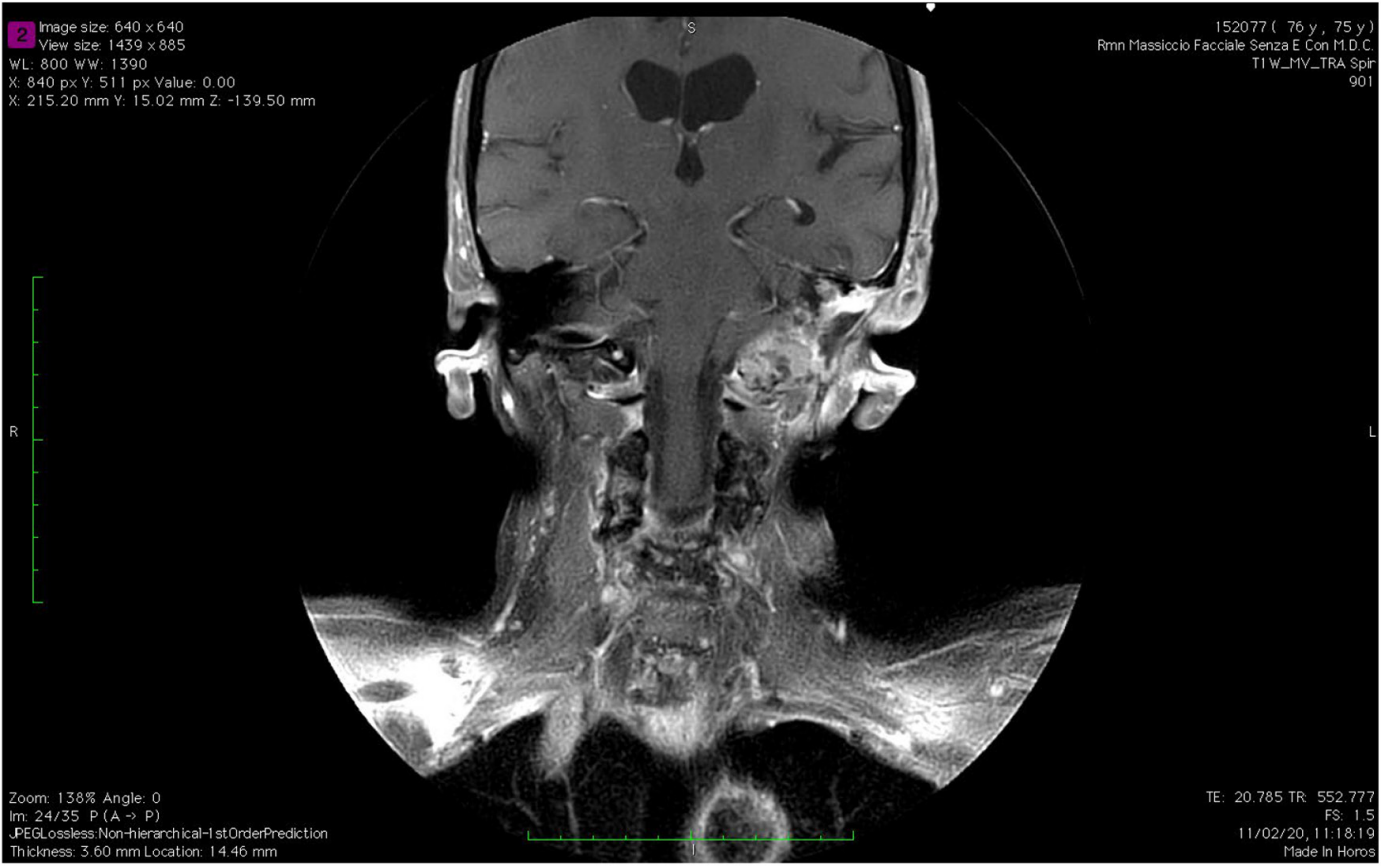
Sixteen months MRI follow up (coronal view). In the site of previous left petrosectomy the MRI scan showed an heterogeneously enhanced tissue; toward the rear, it grows through the foramen lacerum involving the foramen magnum.

**FIGURE 4:**
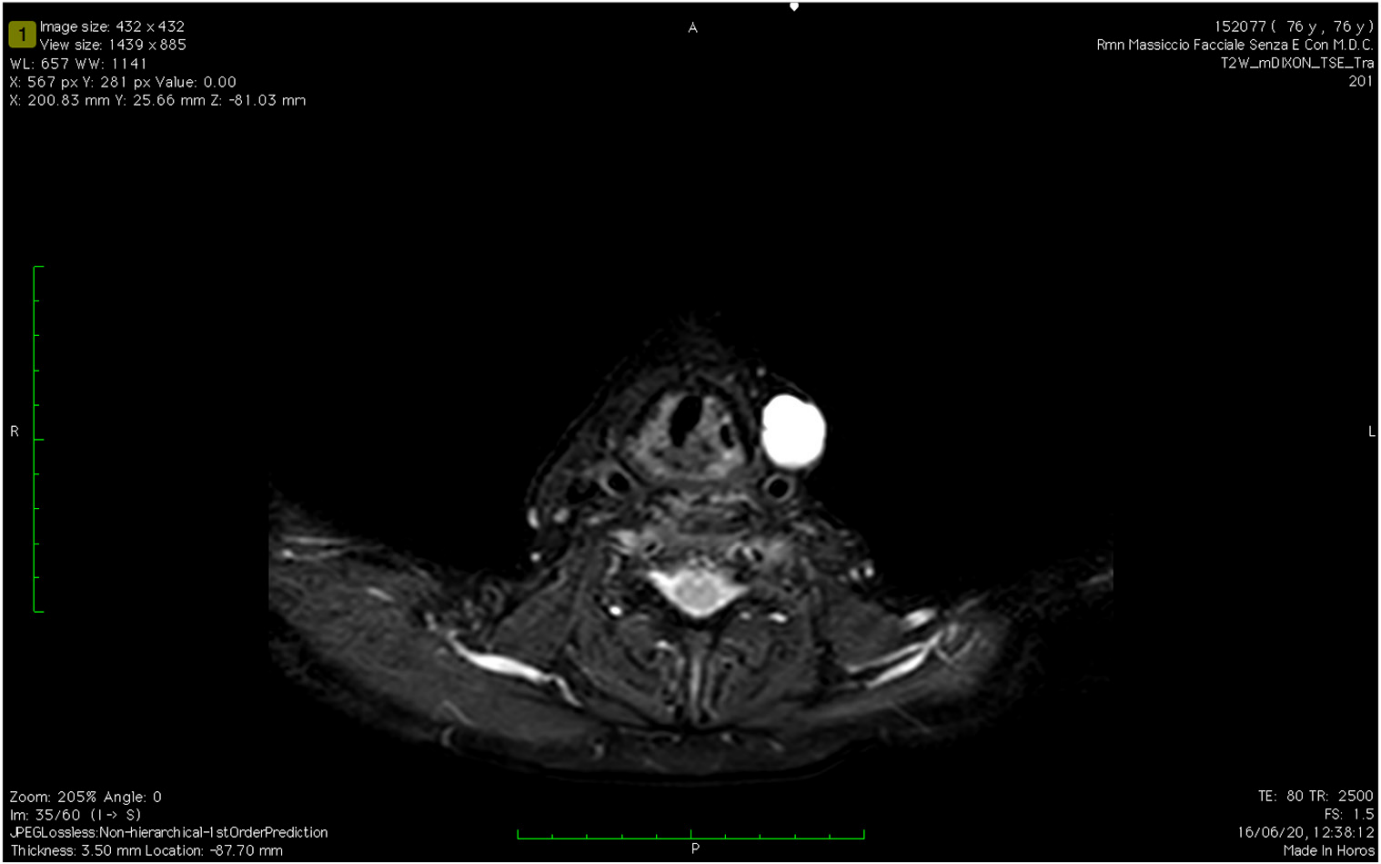
Sixteen months MRI follow up (axial view). Non‐enhancing ovalar mass (2 × 2 cm) in the left thyrohyoid membrane.

At a recent follow‐up, 10 days ago, more than 16 months after surgical revision, an MRI with contrast showed, in the site of previous left petrosectomy, an heterogeneously enhanced tissue growing through the foramen lacerum and involving the foramen magnum (Figure 3). Therefore, the MRI scan showed a non‐enhancing ovalar mass (2 × 2 cm) in the left thyrohyoid membrane, and left retroauricolar lymph nodes with coagulative necrosis (Figure 4). We suggested a decompressive surgery, but the patient refused surgery and decided for hospice care.

## 3 DISCUSSION

Considered benign for up to a few decades, AciCC of the parotid gland has recently been classified as a malignant tumor. Due to its rarity, AciCC has received less attention than other salivary neoplasms.

Diagnosis of AciCC is frequently complicated, due to its similarity with benign tumors. The differential diagnosis is considered, fundamentally, with clear cell carcinomas, mucoepidermoid carcinomas, Warthin's tumor, and oncocytomas. The greatest diagnostic challenge is to differenziate ACC from mammary analogue secretory carcinoma (MASC), a new entity among salivary glands malignancy introduced in the 4th edition of the WHO classification of head and neck tumors.^16^

There are no clear characteristics of AciCCs found on CT, MRI, and ultrasound imaging.

Management of AciCC, especially when metastatic, is very challenging: because of the rarity, data on management are based only on personal clinical experience and retrospective series. The preferred treatment of local disease is complete surgical excision, consisting in total parotidectomy.

The role of radiotherapy remains controversial: the precise indications and oncologic effects of adjuvant radiotherapy in AciCC of the parotid gland are not well known, particularly in patients with negative, but close (<1 mm) margins without other high‐risk histopathologic factors.

According to the most recent literature, the main factors that may predict poor outcomes are (1) extracapsular extension, (2) facial nerve involvement, (3) positive surgical margins, (4) cellular atypia, (5) high pathologic grade as evidenced by >2 mitoses/10 high power fields, (6) necrosis, (7) perineural and vascular invasion, (8) desmoplastic stromal reaction. To our knowledge, only 12 cases of AciCC of the parotid gland with lateral skull base involvement have been reported in literature.

Breen JT et al.^11^ described a unique series of 10 consecutive patients with primary or recurrent AciCC of the parotid gland with skull base invasion or metastasis. After developing skull base disease, four patients underwent surgery, four stereotactic radiosurgery and three external beam radiation. The 10‐year Kaplan–Meyer estimated overall survival after initial diagnosis of parotid AciCC was 80%; after lateral skull base invasion, 2‐year estimated overall survival was 50%.

Buiret G et al.^12^ reported a case of a 62‐year‐old woman with AciCC and skull base invasion treated by external 3D conformational radiation. The tumor showed a regular regression over 2.5‐year period without side‐effects of radiation. They suggested that exclusive external radiation therapy could be an option in case of contra‐indication for surgery or patient refusal.

A study by Douglas JG et al.^13^ suggested that patients with salivary neoplasms with lateral skull base invasion should be considered for a Gamma knife boost after primary treatment with neutron radiotherapy; however, among the patients considered by the study, only one has AciCC with skull base involvement.

The patients described had the following characteristics (we previously excluded the patient from the study of Douglas JG et al., because the authors did not report any data) (Table I); men to women patient ratio was approximately 1:1. Median age was 52.9 years (range, 18–79), and only two patients (18.2%) had skull base disease at initial presentation. Six patients (54.5%) underwent total parotidectomy while three (27.2%) had superficial parotidectomy; in other two patients (18.2%), it is not reported the type of initial surgery. After surgery, only two patients (18.2%) showed positive excision margins; therefore, two patients (18.2%) underwent adjuvant radiotherapy.

**TABLE I:** Characteristics of the patients reported in the studies included in the review.

Study	Patient, gender, age at diagnosis (years)	Surgical approach)	Margins at initial excision	Initial adjuvant therapy	Skull base involvement at initial diagnosis	Time to development of skull base disease (years) and localization	Skull base disease treatment	Positive cervical lymph nodes / Distant metastases
Breen JT et al.^11^	1, F, 18 years	Total parotidectomy	R1	None	No	12; Glenoid fossa, infratemporal fossa, Foramen ovale, Meckel's cave, cavernous sinus, sphenoid sinus, clivus, cerebellum	Extended parotidectomy, partial mandibulectomy, fibular free flap (FFF), external beam and stereotactic radiation	− / +
	2, M, 76 years	Superficial parotidectomy	DNA	None	No	10.1; Masticator space and temporal fossa	External beam radiation	+ / −
	3, F, 23 years	Superficial parotidectomy	R0	None	No	47.2; Parapharyngeal space and petrous temporal bone	LTBR, partial mandibulectomy, FFF, developed subsequent recurrence at petrous temporale bone and clivus	− / +
	4, M, 49 years	Superficial parotidectomy	R0	None	No	14.3; stylomastoid foramen, EAC	Revision parotidectomy, LTBR, external auditory canal resection, SCM flap.	+ / −
	5, F, 79 years	Total parotidectomy	R0	RT	No	4; stylomastoid foramen and jugular foramen	None	− / −
	6, M, 55 years	Total parotidectomy	R0	RT	Yes	DNA; EAC, petrous temporal bone and mastoid, posterior cranial fossa	Subtotal petrosectomy, removal of sigmoid sinus and adjacent dura, trapezius flap, stereotactic radiation	− / +
	7, M, 61 years	None	DNA	DNA	Yes	DNA; On original presentation —TMJ, parapharyngeal space, foramen ovale	None	+ / +
	8, M, 72 years	Total parotidectomy	R0	None	No	10.3; Sphenoid sinus, cavernous sinus, clivus, parietal bone	Stereotactic radiation	− / −
	9, F, 39 years	Parotidectomy of unknown extent	DNA	None	No	36.5; stylomastoid foramen	None	− / −
	10, F, 48 years	Total parotidetomy	R0	None	No	15.3; stylomastoid foramen	Stereotactic radiation	− / −
Buiret G et al.^12^	11, F, 62 years	Total parotidectomy	R0	None	No	8; inferior side of the temporal bone with conserved inner and middle ear and brain structures ad no meningeal involvement	Stereotactic radiation, revision surgery	+ / −
Douglas JG et al.^13^	12, DNA, DNA	DNA	DNA	DNA	DNA	DNA, DNA	Gamma knife boost	DNA

The mean time to develop lateral skull base disease was 15.8 years (range, 4–47.2) (of two patients are not reported these data). This recurrence was treated by surgery alone in two cases (18.2%), with radiotherapy alone in three cases (27.3%), and with revision surgery followed by radiation therapy in three cases (27.3%); for three patients (27.3%) this item is not specified. No patient was treated with chemotherapy regime, confirming the lack of utility of chemotherapy against this histological type of parotid cancer.

Four patients (36.4%) had distant metastases, while other four (36.4%) had cervical metastases. Most frequent skull base localizations were: stylomastoid foramen (4 patients, 36.4%), petrous temporal bone (3 patients, 27.3%), clivus (three patients, 27.3%), foramen ovale (2 patients, 18.2%), cavernous sinus (2 patients, 18.2%), sphenoid sinus (2 patients, 18.2%).

## 4 CONCLUSION

Knowledge about AciCC has changed over the past few decades. Surgery is the first‐line treatment, while radiotherapy can be helpful in selected patients (as patients with positive surgical margins) and in case of recurrence. No chemotherapy regimen is currently being validated.

From the literature, it emerges that a small number of patients will develop lateral skull base involvement, often years after the treatment of the primary disease. These subjects with cranial base disease are at a high risk for multiple late recurrences and even death.

Although in literature there are no clear evidences about neurotrophicity of AciCC of the parotid gland, in our opinion, very rarely these tumors can grow up through the cranial nerves sheath developing within the posterior cranial fossa.

## AUTHOR CONTRIBUTIONS

**Pietro De Luca:** Conceptualization; data curation; formal analysis; funding acquisition; investigation; methodology; resources; validation; visualization; writing – original draft. **Matteo Calvanese:** Data curation; formal analysis; writing – original draft. **Angelo Camaioni:** Formal analysis; supervision; validation; writing – original draft. **Giorgio Iaconetta:** Formal analysis; supervision; validation; writing – original draft. **Maurizio Iemma:** Conceptualization; data curation; formal analysis; supervision; validation; writing – original draft.

## CONFLICT OF INTEREST STATEMENT

The authors have no conflict of interest to disclose. No fundings to declare.

## CONSENT

Written informed consent was obtained from the patient to publish this report in accordance with the journal's patient consent policy.
